# Decreased APE-1 by Nitroxoline Enhances Therapeutic Effect in a Temozolomide-resistant Glioblastoma: Correlation with Diffusion Weighted Imaging

**DOI:** 10.1038/s41598-019-53147-9

**Published:** 2019-11-12

**Authors:** Hye Rim Cho, Nisha Kumari, Nishant Thakur, Hien Thi Vu, Hyeonjin Kim, Seung Hong Choi

**Affiliations:** 1 Department of Radiology, Seoul National University Hospital, Seoul National University College of Medicine, Seoul, 03080 Republic of Korea; 2 Center for Nanoparticle Research, Institute for Basic Science (IBS), Seoul, 00826 Republic of Korea; 3 School of Chemical and Biological Engineering, Seoul National University, Seoul, 00826 Republic of Korea

**Keywords:** Cancer imaging, Cancer imaging

## Abstract

Glioblastoma multiforme (GBM) is one of the most aggressive human tumors with poor survival rates. The current standard treatment includes chemotherapy with temozolomide (TMZ), but acquisition of resistance is a persistent clinical problem limiting the successful treatment of GBM. The purpose of our study was to investigate therapeutic effects of nitroxoline (NTX) against TMZ-resistant GBM *in vitro* and *in vivo* in TMZ-resistant GBM-bearing mouse model, which was correlated with diffusion-weighted imaging (DWI). For *in vitro* study, we used TMZ-resistant GBM cell lines and evaluated therapeutic effects of NTX by clonogenic and migration assays. Quantitative RT-PCR was used to investigate the expression level of TMZ-resistant genes after NTX treatment. For *in vivo* study, we performed 9.4 T MR imaging to obtain T2WI for tumor volume measurement and DWI for assessment of apparent diffusion coefficient (ADC) changes by NTX in TMZ-resistant GBM mice (*n* = 8). Moreover, we performed regression analysis for the relationship between ADC and histological findings, which reflects the changes in cellularity and apurinic/apyrimidinic endonuclease-1 (APE-1) expression. We observed the recovery of TMZ-induced morphological changes, a reduced number of colonies and a decreased rate of migration capacity in TMZ-resistant cells after NTX treatment. The expression of APE-1 was significantly decreased in TMZ-resistant cells after NTX treatment compared with those without treatment. In an *in vivo* study, NTX reduced tumor growth in TMZ-resistant GBM mice (*P* = 0.0122). Moreover, ADC was increased in the NTX-treated TMZ-resistant GBM mice compared to the control group (*P* = 0.0079), which was prior to a tumor volume decrease. The cellularity and APE-1 expression by histology were negatively correlated with the ADC value, which in turn resulted in longer survival in NTX group. The decreased expression of APE-1 by NTX leads to therapeutic effects and is inversely correlated with ADC in TMZ-resistant GBM. Therefore, NTX is suggested as potential therapeutic candidate against TMZ-resistant GBM.

## Introduction

Glioblastoma multiforme (GBM) is one of the most devastating primary malignant brain tumors and remains a major treatment challenge in oncology^1^. Surgery, radiotherapy and adjuvant chemotherapy with temozolomide (TMZ) have been commonly used as the standard treatment^2^. Among them, TMZ, which contain a methylated MGMT promoter, is considered as the most effective chemotherapeutic agent and improves the survival of patients; it is associated with 2-year survival rates of 46% as compared to 14% in patients containing the unmethylated MGMT promoter^3^. However, because of the case of unmethylated MGMT, acquired TMZ-resistance is a major limitation in the treatment of brain tumors and at least, 50% of TMZ-treated patients do not respond to TMZ^4^. To overcome TMZ-resistance, various studies have reported the mechanism of development of resistance to TMZ^5,6^. One study showed that the DNA base excision repair enzyme, APNG confers resistance to TMZ and the downregulation of APNG using siRNA increased the TMZ sensitivity in several established as well as primary GBM cell lines. The authors also showed that the presence of APNG is associated with poor survival in patients and that the loss of APNG improves the survival in xenograft models. Another study reported that the use of a small molecule BER inhibitor potentiates the TMZ tumor cell killing and strongly sensitizes glioma cells to TMZ. Bertucci *et al*. used the mesoporous silica nanoparticles, decorated with polyarginine-peptide nucleic acid to target miRNA, loaded with TMZ and showed a strong induction of apoptosis in TMZ-resistant glioma cells^7^.

Nitroxoline (NTX), is an FDA-approved antibiotic against urinary tract infections that has been repurposed for cancer treatment and could have anti-cancer effects in patients with various kinds of cancer, including leukemia, lymphoma, bladder cancer and pancreatic cancer^8,9^. Mirkovic *et al*. reported the NTX as a reversible inhibitor of cathepsin B, a protein responsible for tumor invasion and showed that NTX inhibited the extracellular and intracellular degradation of the extracellular matrix^10^. Furthermore, another study demonstrated that the NTX induces apoptosis *in vitro* and inhibits the glioma growth in genetically engineered Pten/Kras mice *in vivo*^11^. Since NTX is already being used in humans for UTIs with tolerable side effects, it has the capability to be used against GBM.

Diffusion-weighted imaging (DWI) is a promising technique to evaluate the response to treatment in tumors in both clinical and research settings. The apparent diffusion coefficient (ADC) measured from DWI is sensitive to the tumor microenvironment^12^ and is a potential noninvasive biomarker for the prediction and monitoring of the response to therapy^13^. ADC maps can provide valuable information as they reflect the mobility of water molecules within the tissues and can sensitively detect changed cellularity in tumors^14^. Sugahara *et al*. reported that the ADC is negatively correlated with the tumor cellularity, with a high cell density and extracellular tortuosity resulting in an increased restriction of the diffusion of water molecules^15^.

Considering all these findings, our purpose was to investigate the therapeutic effects of NTX against TMZ-resistant GBM *in vitro* and *in vivo* in a TMZ-resistant GBM bearing mouse model, which was correlated with DWI.

## Results

### NTX treatment recovers cell morphology change and inhibits colony formation and migration in TMZ-resistant GBM cells

**Figure 1:**
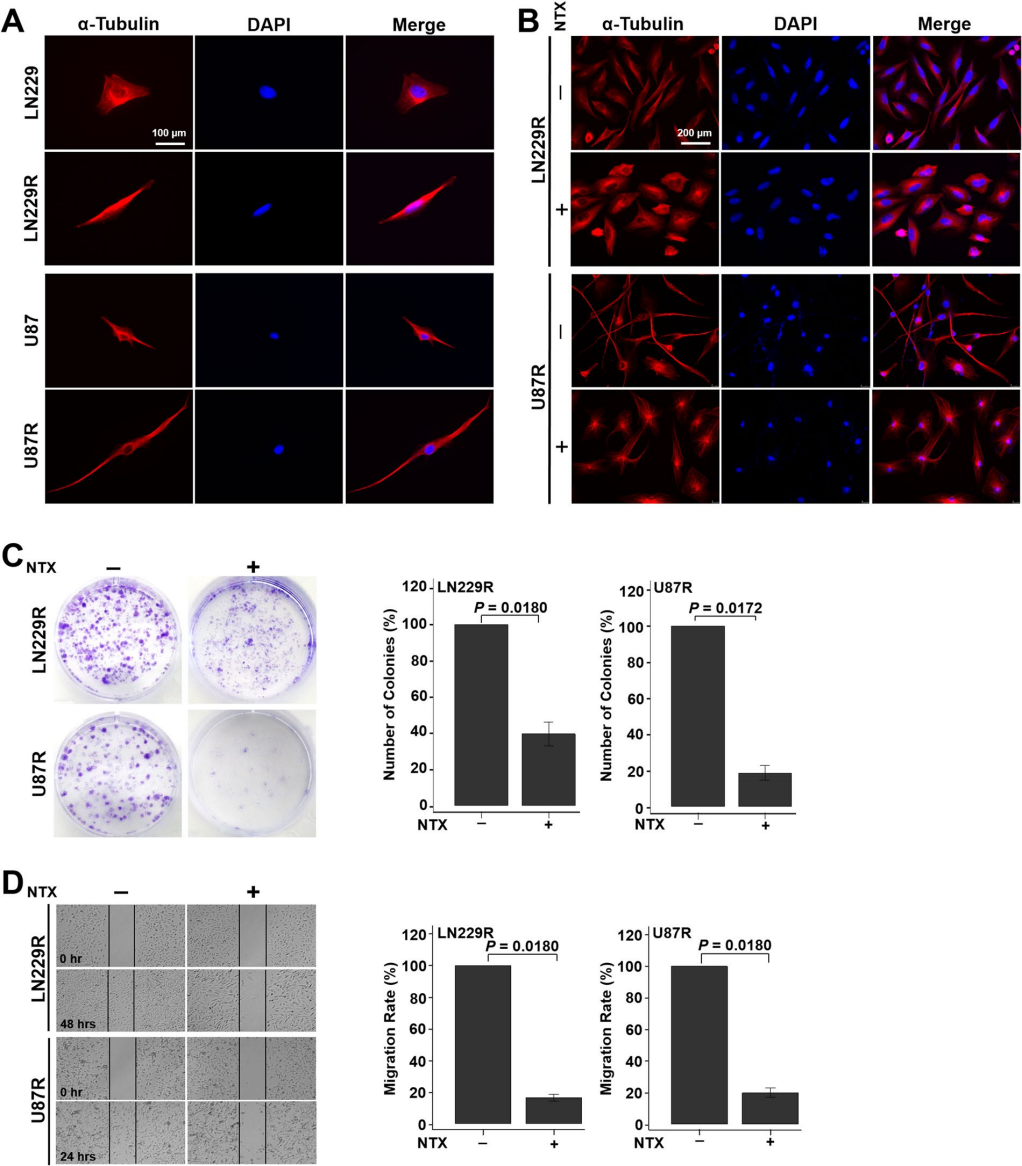
NTX restored changes in cell morphology and inhibited colony formation and migration in TMZ-resistant GBM cells. (**A**) Immunofluorescence images of TMZ-resistant GBM cells and their respective parental cells to compare the morphological differences before and after TMZ treatment. The parental cells changed their morphology from a round or oval shape to a dendritic shape after TMZ treatment in both cell lines. Magnification: ×400. (**B**) The morphology of LN229R and U87R cells recovered as parental cells after NTX treatment. Magnification: ×200. (**C**) Colony formation was significantly decreased in LN229R (*P* = 0.0180) and U87R (*P* = 0.0172) cell lines after NTX treatment. (**D**) The migration capacity was also significantly reduced in LN229R after 48 hours (*P* = 0.0180) and in U87R after 24 hours (*P* = 0.0180) after NTX treatment. Note: LN229R = TMZ-resistant LN229 and U87R = TMZ-resistant U87.

TMZ-resistance induced morphological transformations in both of the resistant cell lines as the cells changed from round or oval shapes to elongated and spindle shapes, as demonstrated by α-tubulin staining under a fluorescence microscope (Fig. 1A). However, the cytoskeleton was disrupted and recovered to the original parental morphology after 10 µg/ml of NTX treatment for 24 hours in both of the TMZ-resistant cell lines (Fig. 1B). To evaluate the therapeutic effects of NTX *in vitro*, we performed a colony formation and migration assay. NTX (0.5 µg/ml) significantly reduced the formation of colonies in both of the TMZ-resistant cell lines after 10 days of treatment. Approximately 60% and 80% of the colonies were inhibited in TMZ-resistant LN229 (LN229R) (*P* = 0.0180) and TMZ-resistant U87 (U87R) (*P* = 0.0172), respectively (Fig. 1C). In a migration assay, we found that 10 µg/ml of NTX inhibited approximately 84% and 80% of the migration over 48 hours in LN229R (*P* = 0.0180) and over 24 hours in U87R (*P* = 0.0180), respectively, when compared to their respective nontreated groups (Fig. 1D).

### NTX reduces APE-1 expression in TMZ-resistant GBM cells

**Figure 2:**
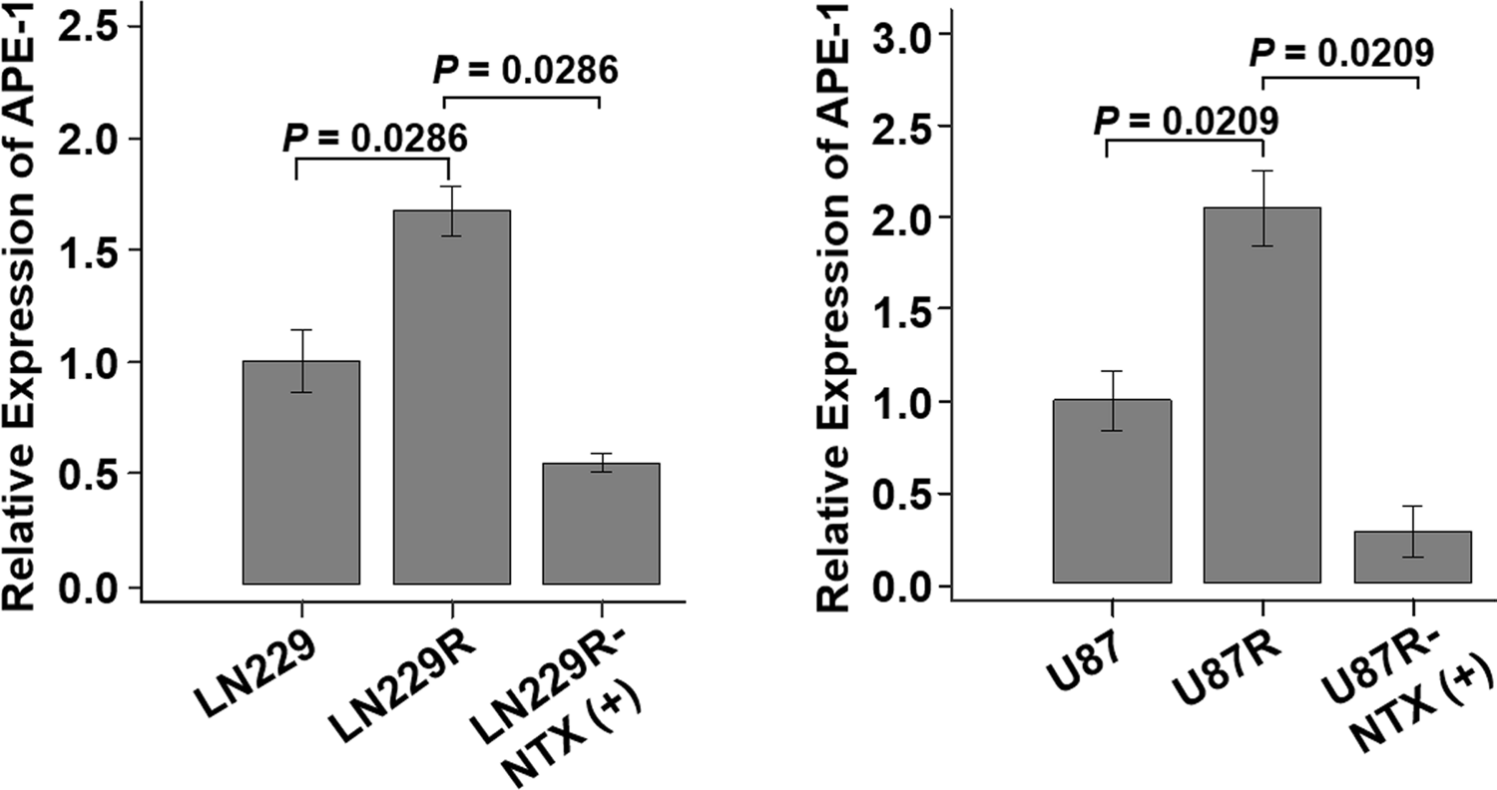
Analysis of APE-1 expression after NTX treatment. The relative expression of APE-1 was significantly increased in TMZ-resistant cell lines (LN229 vs LN229R, *P* = 0.0286 and U87 vs U87R, *P* = 0.0209) compared to the parental cell lines, which was significantly decreased after NTX treatment (LN229R vs LN229R-NTX, *P* = 0.0286 and U87 vs U87R-NTX, *P* = 0.0209) in both TMZ-resistant cell lines. Note: LN229R = TMZ-resistant LN229 and U87R = TMZ-resistant U87.

Based on the *in vitro* results, we selected several genes [APE-1, MutS protein homolog 2 (MSH-2), MutS homolog 6 (MSH-6), O^6^-alkylguanine DNA alkyltransferase (MGMT) and connexin 43 (Cx43)], all of which are known to be associated with TMZ-resistance, and we compared the expression levels of all the genes (Supplementary Fig. S1). Among them, only APE-1 showed an increased expression with statistical significance in both of the LN229R and U87R cell lines. The relative expression of APE-1 was found to be significantly increased in both TMZ-resistant cell lines compared to their respective parental cells: [LN229: 0.9967 (IQR, 0.9402–1.0654), LN229R: 1.6590 (IQR, 1.6189–1.7294), *P* = 0.0286] and [U87: 0.9700 (IQR, 0.9450–1.0600), U87R: 2.0150 (IQR, 1.9600–2.1450), *P* = 0.0209]. The expression of APE-1 was significantly decreased in both TMZ-resistant cell lines after 24 hours of NTX treatment ([LN229R: 1.6590 (IQR, 1.6189–1.7294), LN229R-NTX: 0.5435 (IQR, 0.5286–0.5686), *P* = 0.0286] and [U87R: 2.0150 (IQR, 1.9600–2.1450), U87R-NTX: 0.3500 (IQR, 0.2500–0.3600), *P* = 0.0209]) (Fig. 2). In addition, an increased expression of cleaved caspase-3 and PARP after NTX treatment was noted in both TMZ-resistant cell lines (Supplementary Fig. S2).

### NTX treatment of TMZ-resistant GBM mouse model increases ADC value and survival

**Figure 3:**
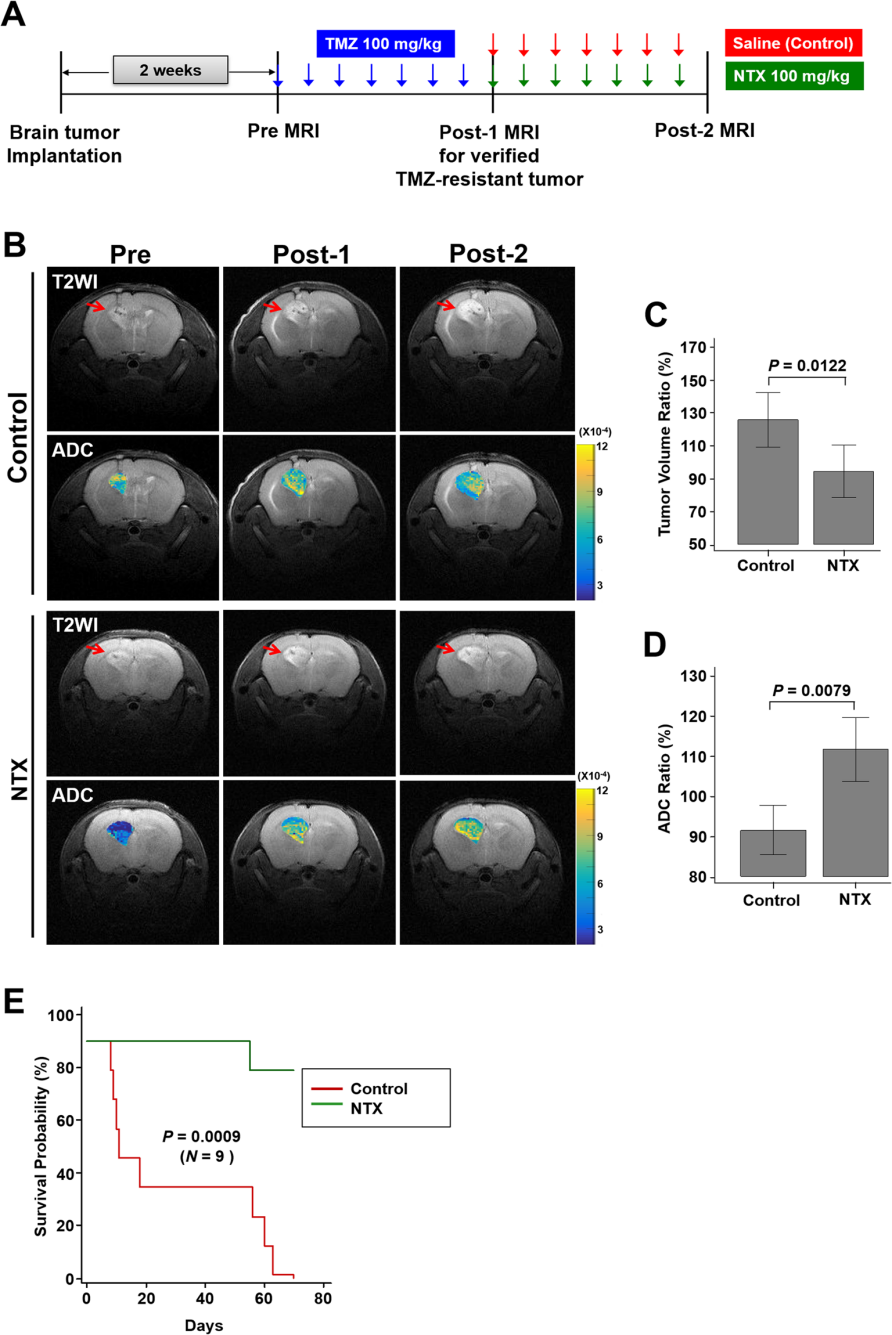
Short-term *in vivo* MR study and long-term survival analysis in a TMZ-resistant GBM mouse model. (**A**) The experimental design for the *in vivo* MR study (*n* = 4, in each group). (**B**) Representative T2WI (1^st^ row) and ADC maps (2^nd^ row) for the control group and T2WI (3^rd^ row) and ADC maps (4^th^ row) for the NTX group, visualized with pretreatment MRI and post-1 MRI to verify TMZ resistance and with post-2 MRI to assess NTX effects. (**C**) The tumor volume ratio (%) was significantly decreased in the NTX group (*P* = 0.0122) compared to the control group. (**D**) The mean ADC ratio was significantly increased (*P* = 0.0079) in the NTX group. (**E**) The survival of the NTX group was significantly longer than that of the control group, which was analyzed by Kaplan-Meier survival analysis (*n* = 9, in each group) [mean, 72.8 days (95% CI, 68.7–76.9) vs 33.9 days (95% CI, 17.1–50.7), *P* = 0.0009, log-rank test].

The *in vivo* MR study with a TMZ-resistant GBM mouse model was performed as shown in Fig. 3A. Figure 3B represents the T2WI for anatomy and ADC maps for the response of treatment in both the control and NTX groups. The tumor volume ratio was significantly lower in the NTX group after 1-week of NTX treatment compared to the control group [control: 128.00% (IQR, 113.25–136.75), NTX: 97.00% (IQR, 87.75–101.25), *P* = 0.0122] (Fig. 3C). Moreover, a greater increase in the ADC ratio was observed in the NTX group than in the control group, which was detectable 1 week after the initiation of NTX treatment [control: 91.40% (IQR, 88.12–96.37), NTX: 110.00% (IQR, 108.00–115.25), *P* = 0.0079] (Fig. 3D). However, there was no significant difference in tumor volume in the NTX group between post-1 and post-2 MRI [post-1: 6.38 mm^2^ (IQR, 5.15–7.54), post-2: 6.03 mm^2^ (IQR, 4.49–7.39), *P* = 0.3071], while the control group showed a significant increase in tumor volume between the two MRI scans [post-1: 4.57 mm^2^ (IQR, 3.39–5.79), post-2: 5.62 mm^2^ (IQR, 4.75–6.48), *P* = 0.0015]. In both the control and NTX groups, a significant decrease and increase in the ADC value were observed between the post-1 and post-2 MRI, respectively ([control: post-1: 8.05 × 10^−4^ mm^2^/sec (IQR, 7.88 × 10^−4^–8.36 × 10^−4^), post-2: 7.37 × 10^−4^ mm^2^/sec (IQR, 7.19 × 10^−4^–7.53 × 10^−4^), *P* = 0.0163] and [NTX: post-1: 7.36 × 10^−4^ mm^2^/sec (IQR, 6.71 × 10^−4^–8.19 × 10^−4^), post-2: 8.23 × 10^−4^ mm^2^/sec (IQR, 7.10 × 10^−4^–9.10 × 10^−4^), *P* = 0.0172]). In the long-term survival study, the NTX group showed a significantly extended survival compared to the control group [mean: 72.8 days (95% CI, 68.7–76.9) vs 33.9 days (95% CI, 17.1–50.7), *P* = 0.0009, log-rank test] (Fig. 3E). However, TMZ had no significant effect on either early response or long-term survival (Supplementary Fig. S3 and Supplementary Table S1).

### APE-1 expression in the TMZ-resistant GBM mouse model was inversely correlated with the ADC value after NTX treatment

**Figure 4:**
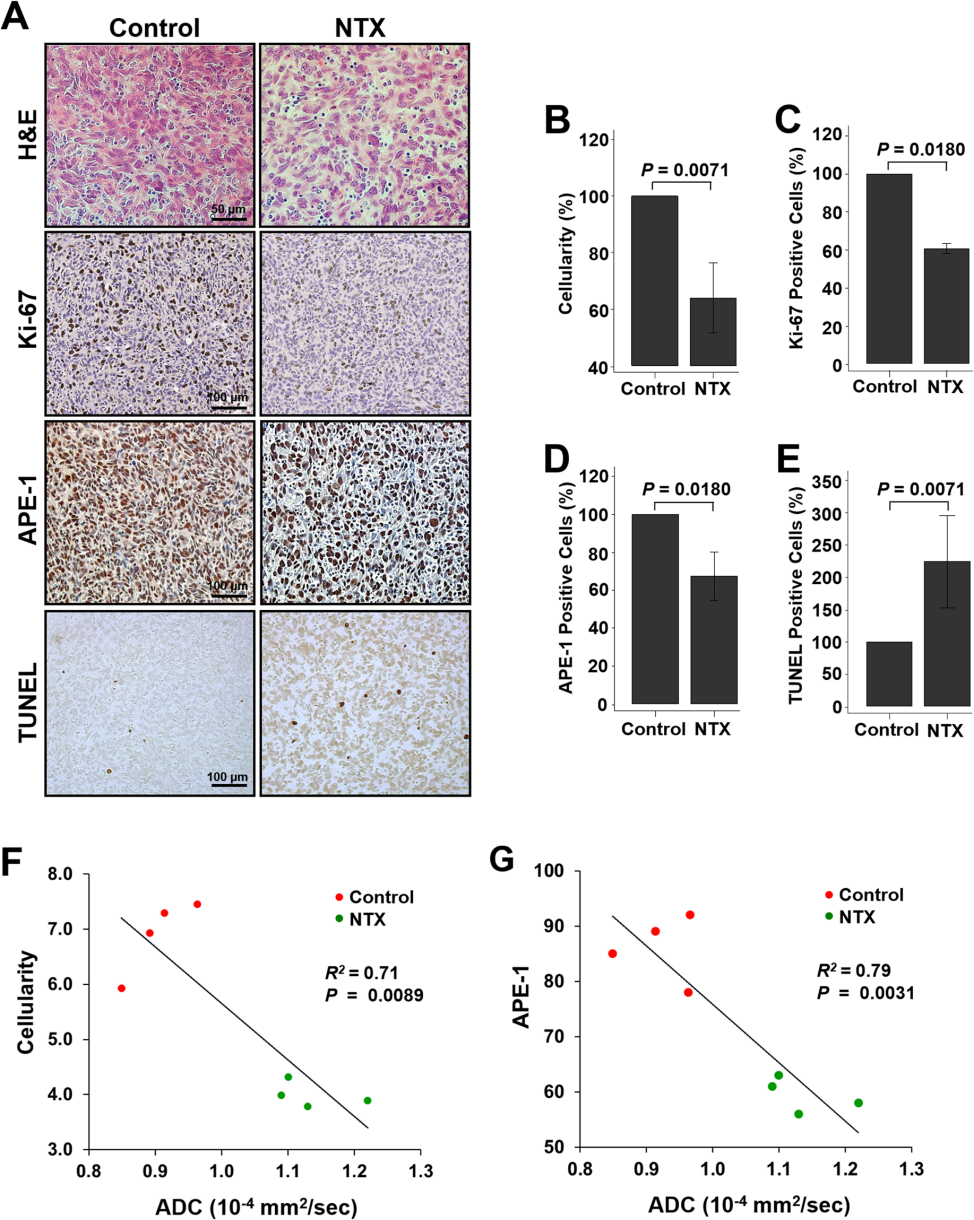
Histological analysis after NTX treatment. (**A**) Histological images representing hematoxylin and eosin (H&E) staining (1^st^ row), immunohistochemistry images of Ki-67, (2^nd^ row), APE-1 (3^rd^ row) and TUNEL staining (4^th^ row) for the control and NTX groups (×400 for H&E and ×200 for immunohistochemistry). (**B**–**D**) The cellularity (*P* = 0.0071) evaluated by H&E, Ki-67 (*P* = 0.0180), and APE-1 (*P* = 0.0180) was significantly decreased in the NTX group compared to the control group. (**E**) Moreover, apoptotic cells, evaluated by TUNEL assay, were significantly increased in the NTX group (*P* = 0.0071). (**F**,**G**) The tumor cellularity and level of APE-1 showed a negative correlation with the ADC value (*P* = 0.0089, *R*^*2*^ = *0*.*71*) and (*P* = 0.0031, *R*^*2*^ = *0*.*79*), respectively, in both the control and NTX groups.

Figure 4A shows the histological analysis. In the case of H&E staining, for morphology and cellularity in tumors, a significant difference was observed in the NTX group compared to the control group [control: 100.00% (IQR, 100.00–100.00); NTX: 66.00% (IQR, 54.75–69.75), *P* = 0.0071] (Fig. 4B). Moreover, the expression levels of Ki-67 [control: 100.00% (IQR, 100.00–100.00), NTX: 61.50% (IQR, 59.00–62.50), *P* = 0.0180] and APE-1 [control: 100.00% (IQR, 100.00–100.00), NTX: 70.00% (IQR, 62.00–73.00), *P* = 0.0180] were significantly decreased in the NTX group compared with the control group (Fig. 4C,D). In the TUNEL assay for investigating DNA damage, a significantly increased number of apoptotic cells was observed after NTX treatment [control: 100.00% (IQR, 100.00–100.00), NTX: 212.00% (IQR, 155.00–247.25), *P* = 0.0071] when compared to the control group (Fig. 4E). These results were validated by histology and agreed with the increased ADC value assessed by DWI. A significant inverse correlation was observed between the cellularity and the level of APE-1 and ADC [cellularity (*R*^*2*^ = 0.71, *P* = 0.0089) and APE-1 (*R*^*2*^ = 0.79, *P* = 0.0031)] in both the control and NTX groups (Fig. 4F,G).

## Discussion

Our study demonstrates that NTX can act as an anticancer compound against TMZ-resistant GBM through the *in vitro* and *in vivo* studies. First, NTX could reduce the expression of APE-1, an essential protein for DNA base excision repair and redox regulation, in TMZ-resistant GBM cells. Second, we observed an increase in the ADC value before a significant tumor volume change soon after NTX treatment in the TMZ-resistant GBM mouse model, which indicates a prior decrease in GBM cellularity before the shrinkage of tumor burden, as well as a long-term survival gain of NTX treatment in the TMZ-resistant GBM mouse model. Third, both APE-1 expression and tumor cellularity were inversely correlated with the ADC value after NTX treatment in the TMZ-resistant GBM mouse model, which suggests that DWI can be used as an imaging surrogate marker of early response to chemotherapy.

We selected several genes related to TMZ-resistance through previous studies^4,16–20^ and investigated regulated the gene levels after NTX treatment in two TMZ-resistant cell lines. Interestingly, the increased APE-1 level of both TMZ-resistant cells compared to the parental cells was significantly decreased in TMZ-resistant cells after NTX treatment. This pattern was consistent with the morphological changes, which showed a recovery of cell morphology to parental cells after NTX treatment in both TMZ-resistant cell lines (Figs 1B and 2). However, other genes including MSH-2, MSH-6, MGMT, and Cx43 did not show similar changes (Supplementary Fig. S1).

APE-1 activity is elevated in human gliomas and confers resistance to ionizing radiation and alkylating agents^21,22^. Demple *et al*. reported the interfered cell proliferation by the strong downregulation of APE-1 in cancer cell lines, which leads to the activation of apoptosis^23^. Related to these reports, our data showed an increased expression level of cleaved caspase-3 (CC3) and cleaved PARP, which are associated with the concentration of NTX in both TMZ-resistant cell lines (Supplementary Fig. S2). Previous reports have revealed that the cytoskeleton is composed of α- and β-tubulin, which form the microtubules that are required for cell differentiation and the maintenance of structural integrity^24^. In addition, the migrative and invasive properties of GBM cells were found to be potentially blocked by a microtubule inhibitor^25^. It is also obvious that the migrative and invasive features of GBM cells contribute to the development of resistance to apoptosis^26,27^. In agreement with previous reports, by the use of immunofluorescence staining, we found that NTX disrupted the microtubule structures of both TMZ-resistant GBM cell lines, leading to impairment of the colony formation capacity and the migrative and invasive properties of cancer cells. Based on these findings, the downregulated APE-1 by NTX was able to increase the therapeutic effect against TMZ resistance, which can be supported by the reduced colony formation and migration capacities after NTX treatment as well (Fig. 1).

To investigate the therapeutic effect of TMZ resistant GBM by NTX *in vivo*, we developed a TMZ-resistant GBM-bearing mouse model^4,28,29^ and assessed cellularity changes using the ADC parameter^30^, respectively. In our study, the TMZ-resistant GBM-bearing mouse model was confirmed by investigating the tumor size and ADC value after TMZ treatment for 1 week. All control, NTX, and TMZ groups showed an increased tumor size which indicates the development of resistance even after TMZ. In our present study, to assess the early therapeutic effect of NTX against TMZ-resistant GBM, we measured changes in tumor volume and ADC values, where we observed a more prominent ADC increase of TMZ-resistant GBM in the NTX group than control group, which was prior to the tumor volume decrease. We also confirmed the long-term survival gain of NTX in the TMZ-resistant GBM model as well. These results suggest that NTX has a potent anti-tumor activity even within the early period, which resulted in the extended survival of the TMZ-resistant GBM model. One might assume that the 1-week TMZ exposure may be insufficient to develop a TMZ-resistant GBM model. To further scrutinize this possibility, we investigated the TMZ group, which was given TMZ for one more week after the confirmation of a TMZ-resistant GBM-bearing mouse model (total time TMZ received: 2 weeks), and we also observed subsequent increased tumor volume and decreased ADC values after persistent TMZ treatment (Supplementary Fig. S3). This finding clearly reflects that GBM had further progressed as resistance developed to TMZ in the TMZ group^31,32^.

The ADC changes after NTX treatment were also correlated with the histological findings including significantly decreased cellularity and cell proliferation in the NTX group of the TMZ-resistant GBM model compared with the control group (Fig. 4), which are, in particular, supported by the decreased APE-1 expression level and the increased TUNEL-positive cells, well correlated with the *in vitro* results in our study (Figs 2 and 4 and Supplementary Fig. S2). Moreover, numerical data of cellularity change after NTX treatment by H&E which were consistent with a previous report^33^ and the APE-1 expression change by immunohistochemistry were negatively correlated with the ADC value in both the control and NTX groups (Fig. 4F,G). This demonstrated that the ADC value could be used as a noninvasive biomarker to evaluate the early treatment effect of NTX in TMZ-resistant GBM, which could result in long-term survival gain.

Our study has a limitation. In a TMZ-resistant GBM mouse model, we used a limited number of mice in each group for the evaluation of an early response to NTX as well as long-term survival to minimize the animal number according to the guideline of the institutional animal care and use committee in our institute.

In conclusion, we have demonstrated that the NTX could induce apoptosis through the decreased expression of APE-1 in TMZ-resistant GBM *in vitro* and inhibit the growth of a TMZ-resistant GBM tumor model, which is confirmed by a decreased tumor volume and an increased ADC after short-term NTX therapy in addition to extended survival. Particularly, since MGMT is overexpressed in ~70% of GBM, our study supports that NTX is a promising candidate for future clinical trials against TMZ-resistant GBM as a second-line treatment regimen.

## Materials and Methods

### Cell lines and Drugs

The human GBM cell lines LN229 and U87MG were purchased from the American Type Culture Collection (ATCC) and used for our study. LN229 and U87MG were cultured in DMEM and RPMI supplemented with 10% fetal bovine serum (FBS) and 1% penicillin/streptomycin at 37 °C in the presence of 5% CO_2_. TMZ-resistant cell lines were established from the parental cells (LN229 and U87) by continuously exposing them to low doses of TMZ and designated LN229R and U87R for our study.

NTX and TMZ were obtained from Sigma Aldrich and dissolved in dimethyl sulfoxide (DMSO) to prepare stock concentrations, which were further diluted with culture medium to working concentrations as required.

### TMZ-resistant GBM mouse model

This study was approved by the institutional animal care and use committee of the Seoul National University Hospital. All research was performed in accordance with the relevant guidelines/regulations in our institute (16-0130-C1A0). To prepare the TMZ-resistant GBM mouse model, 6-week-old male BALB/c nude mice (*n* = 8) were anesthetized by the intraperitoneal injection of a mixture of Zoletil (zolazepam) and Rompun (xylazine) and placed in a stereotaxic device. LN229 GBM cells (3 × 10^6^ cells) were injected into the caudate/putamen region of the brain by using a Hamilton syringe fitted with a 28-gauge needle. Two weeks after tumor implantation, the required tumor size was confirmed by pretreatment MRI. For TMZ-resistant models, GBM-bearing mice were developed by administering successive high doses of TMZ (100 mg/kg/day) intraperitoneally until tumor growth showed no inhibition by TMZ for 7 days, as described in previous studies^4,28,29^. The models were further confirmed by post-1 MRI and T2WI, which is an MR protocol that was used for the assessment of tumor size.

### Short-term *in vivo* treatment response study in a TMZ-resistant GBM mouse model

For the *in vivo* animal MRI, the animals were anesthetized with 1.5–2% isoflurane/oxygen (v/v), and then scanned by using a 9.4T MR scanner (Agilent Technologies, Santa Clara, CA, USA). Throughout each imaging session, the animals were wrapped in warm water blankets, and their oxygen saturation and heart rates were monitored. For the short-term treatment response study, we divided the animals into 2 groups after the confirmation of TMZ-resistance by post-1 MRI. Four TMZ-resistant GBM mice were intraperitoneally injected with 1% saline for 7 days (control group). The remaining four TMZ-resistant GBM mice were intraperitoneally treated with 100 mg/kg/day of NTX for 7 days (NTX group). The post-2 MRI was conducted after the saline or NTX treatment to evaluate the therapeutic effects on tumor growth. DWI was also acquired sequentially.

### MRI protocol and analysis

In the T2WI in the coronal plane, fast spin-echo multi-slices methods were employed (TR n 3000 ms, effective TE = 31.18 ms, ETL = 4, average = 2, data matrix size = 256 × 256, and FOV (field of view) = 25.0 × 25.0 mm^2^). The echo-planar DWI in the coronal plane was also obtained (TR/TE = 4000/60.04 ms, with shots = 2, repetitions = 1, average = 2, matrix = 128 × 128, FOV = 24.0 × 24.0 mm^2^, b-value = 0, 100, 200, 400, 700 and 1000 s/mm^2^, 1 mm slice thickness). In addition, apparent diffusion coefficient (ADC) maps were generated, and image analysis was performed by using our in-house software developed with a commercial analysis package (MATLAB version R2007b, MathWorks Inc., Natick, MA, USA).

One investigator (N. K.) who was blinded to the experimental data drew the regions of interest (ROIs) that contained the entire tumor on every continuous section of the coregistered T2WI and ADC maps. Tumor boundaries were defined with reference to the high-signal intensity areas thought to represent tumor tissue on the T2WI. Finally, we calculated the tumor volume and mean ADC for each tumor, as well as the ratios of the tumor volumes and the ADC values (post-2 value/post-1 value × 100).

### Long-term survival analysis in a TMZ-resistant GBM mouse model

For the long-term survival analysis of 70 days, we used TMZ-resistant GBM mice (*n* = 18) generated by a previously described method. The mice were divided into two groups [control, (*n* = 9, saline-treated) and NTX, (*n* = 9)] according to tumor sizes, which were equally distributed in each group. The saline solution or NTX were injected in the same manner as described above in the control and NTX groups, respectively, during the follow-up period. All mice were observed until euthanasia or the survival endpoint of 70 days.

### Immunohistology

For immunohistological analysis, all animals were sacrificed at the end of the MRI studies. The brain tissues were extracted, preserved in 10% formalin and embedded in paraffin using standard protocols. We prepared 4-µm thick tissue sections and then stained them with APE-1 (Novus Biologicals; for DNA-repair) and Ki-67 (UltraMab; for cancer cell proliferation) as primary antibodies. Then, the sections were rinsed with washing buffer and incubated with secondary antibodies (Santa Cruz Biotechnology), then evaluated by DAB. The APE-1- and Ki-67-positive cells were analyzed by using ImageJ software. Additionally, the TUNEL assay was used to measure apoptotic cells.

### Statistical analysis

All statistical analyses were performed using MedCalc Software (MedCalc version 13.1.0.0). The Kolmogorov-Smirnov test was used to determine the normal distribution of the noncategorical variables. The nonparametric data are presented as the median and interquartile range (IQR, range from the 25^th^ to the 75^th^ percentile), and the parametric data are shown as the mean ± standard deviation. According to the results of the Kolmogorov-Smirnov test, a paired or unpaired Student’s t-test, Mann-Whitney U-test or Wilcoxon test was performed, as appropriate, to compare the values between two groups. For correlation studies, we used Spearman’s correlation. Survival data in the GBM mouse model were analyzed by using the Kaplan-Meier with a log rank method and the Cox proportional hazards model in the GBM mouse model.

## Supplementary information

Supplementary Information
