# Supplementary material for: Decreased APE-1 by Nitroxoline Enhances Therapeutic Effect in a Temozolomide-resistant Glioblastoma: Correlation with Diffusion Weighted Imaging
Source: Sci Rep. 2019 Nov 12;9:16613. doi: 10.1038/s41598-019-53147-9 (PMC6851184; doi:10.1038/s41598-019-53147-9)
Supplement: Supplementary file 1 — Supplementary Information [file 41598_2019_53147_MOESM1_ESM.doc]

**Supplementary Material**

**Decreased APE-1 by Nitroxoline Enhances Therapeutic Effect in a Temozolomide-resistant Glioblastoma: Correlation with Diffusion Weighted Imaging**

Hye Rim Cho, Ph.D.^1,2#^, Nisha Kumari, M.S.^1#^, Nishant Thakur, M.S.^1^, Hien Thi Vu, M.S.^1^, Hyeonjin Kim, Ph.D.^1^, Seung Hong Choi, M.D., Ph.D.^1,2,3*^

^1^Department of Radiology, Seoul National University Hospital, Seoul National University College of Medicine, Seoul, 03080, Republic of Korea. ^2^Center for Nanoparticle Research, Institute for Basic Science (IBS), Seoul, 00826, Republic of Korea. ^3^School of Chemical and Biological Engineering, Seoul National University, Seoul, 00826, Republic of Korea.

^#^Hye Rim Cho and Nisha Kumari contributed equally to this study.

**^*^Corresponding Author:** Seung Hong Choi, M.D., Ph.D.

Department of Radiology, Seoul National University College of Medicine

Center for Nanoparticle Research, Institute for Basic Science, and School of Chemical and Biological Engineering, Seoul National University

101, Daehak-ro, Jongno-gu, Seoul, 03080, Korea

Tel: 82-2-3668-7832; Fax: 82-2-747-7418

E-mail: [verocay@snuh.org](mailto:verocay@snuh.org)

**Materials and Methods**

***Immunofluorescence analysis***

TMZ-resistant cells (1x10^5^) were seeded and treated with 10 µg/ml NTX (experimental group) or DMSO (nontreated group) after 24 hours. After 24 hours, the cells were fixed in 4% formaldehyde for 15 minutes at room temperature and permeabilized for 10 minutes in 0.1% Triton X-100. After washing with PBS, the cells were incubated with BSA for 30 minutes for blocking. The cells were incubated with anti-tubulin primary antibody (1:100, Sigma Aldrich) for 1 hour at room temperature. After rinsing with PBS, the cells were stained with a secondary antibody (Alexa Fluor 594 goat anti-mouse IgG, Invitrogen) for 1 hour and mounted. For fluorescence imaging, the cells were observed under a fluorescence microscope (LEICA CTR5500), and all images were captured under the same parameters.

***Colony formation assay***

Colony formation assays were performed by seeding TMZ-resistant cells (1000 cells/2 ml) in 6-well plates. The experimental groups were treated with 0.5 µg/ml NTX, and the control group was supplemented with medium containing TMZ. All groups were observed for 10 days. After 10 days, the cells were fixed with 4% paraformaldehyde and stained with 0.5% crystal violet solution at room temperature. The cell density was evaluated by the crystal violet staining of the adherent cells. The colony formation inhibition rate was calculated using the following formula:

plating efficiency = number of colonies counted / number of cells plated x 100%.

***Migration assay***

The cell migration capacity was investigated using ibidi culture-insert 2-well plates (Ibidi, Bonn). The culture inserts consisted of two wells separated by a 500-mm thick wall. The same amount of cell suspension (5×10^5^ cells/ml) was dispensed in each plate, including each well, followed by incubation in a CO_2_ incubator at 37°C. After approximately 6 to 8 hours, when the cells had attached completely, the cells were gently treated with either 10 µg/ml NTX or culture medium. To investigate the migration capacity of cells, the wall was removed very gently under aseptic conditions and that time point was considered 0 hours. After 24 and 48 hours of treatment, the space between the two cell layers was observed under a microscope. All experiments were carried out in quadruplicate.

***Real-time PCR***

RNA was extracted with TRIzol reagent (Invitrogen). Reverse transcription was performed using the high capacity cDNA reverse transcription kit (Life Technologies-Applied Biosystems) in accordance with the manufacturer’s recommendations. Real-time PCR was performed with 1 µg of cDNA on a CFX Connect Real-Time PCR (BIORAD) using iQ SYBR green. The initial incubation was at 65 ºC for 10 minutes. The primer sequences used in the PCR were as follows: Apurinic/apyrimidinic endonuclease (APE-1): (F) 5´-TTGTGGCTGAATTTGACTCG-3´ (R) 3´-TTGAGGTCTCCACACAGCAC-5´, MutS protein homolog 2 (MSH-2): (F) 5´-GCCATTTTGGAGAAAGGACA-3´ (R) 3´-CTCACATGGCACAAAACACC-5´, MutS protein homolog 5 (MSH-6): (F) 5´-GATTCCATTGGGTTGACACC-3´ (R) 5´-AGCACCATTCGTTGATAGGC-3´, O^6^-alkylguanine DNA alkyl transferase (MGMT): (F) 5´-GTGGCCTCACGATGTGTATG -3´ (R) 3´-ACCCGCTGTGTGACTTATCC-5´ and connexin 43 (Cx43): (F) 5´-ATGAGCAGTCTGCCTTTCGT-3´ (R) 3´-TCTGCTTCAAGTGCATGTCC-5´. All primers were purchased from Bioneer. The relative expression was calculated using 2 (-Delta Delta CT).

***Western Blot***

The TMZ-resistant cells **(**1x10^5^) were seeded and treated with different concentrations of NTX for 24 hours. For western blot analysis, 50 μg/ml of proteins were loaded onto the gel, and the following primary antibodies were used: anti-cleaved caspase-3 (Cell Signaling Technology), anti-poly (ADP-ribose) polymerase (PARP) (Cell Signaling Technology), and anti-β-actin (Abcam). All primary antibodies were detected by horseradish peroxidase-conjugated secondary antibodies (Santa Cruz Biotechnology). The band intensities were quantified using ImageJ software.

**Supplementary Figures**


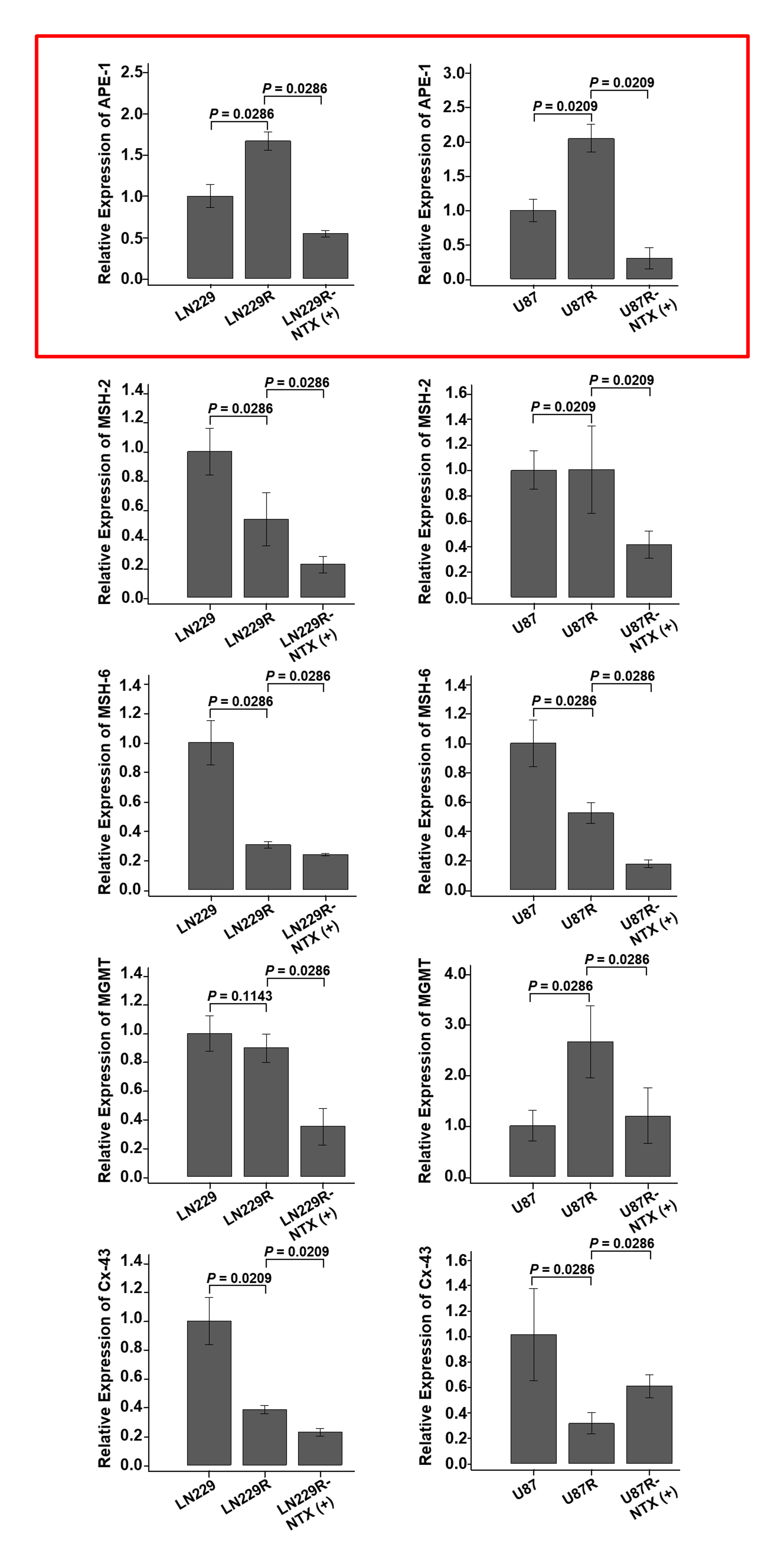


**Figure S1.** Effect of NTX treatment on the expression of genes related to TMZ resistance. The expression of APE-1 was increased in TMZ-resistant cells compared to their parental cells (LN229 vs LN229R: *P* = 0.0286 and U87 vs U87R: *P* = 0.0209), which was found to be significantly decreased after NTX treatment (LN229R vs LN229R-NTX: *P* = 0.0286 and U87R vs U87R-NTX: *P* = 0.0209). These patterns were observed identically in both cell lines only for the expression of APE-1. Note: LN229R = TMZ-resistant LN229, and U87R = TMZ-resistant U87.


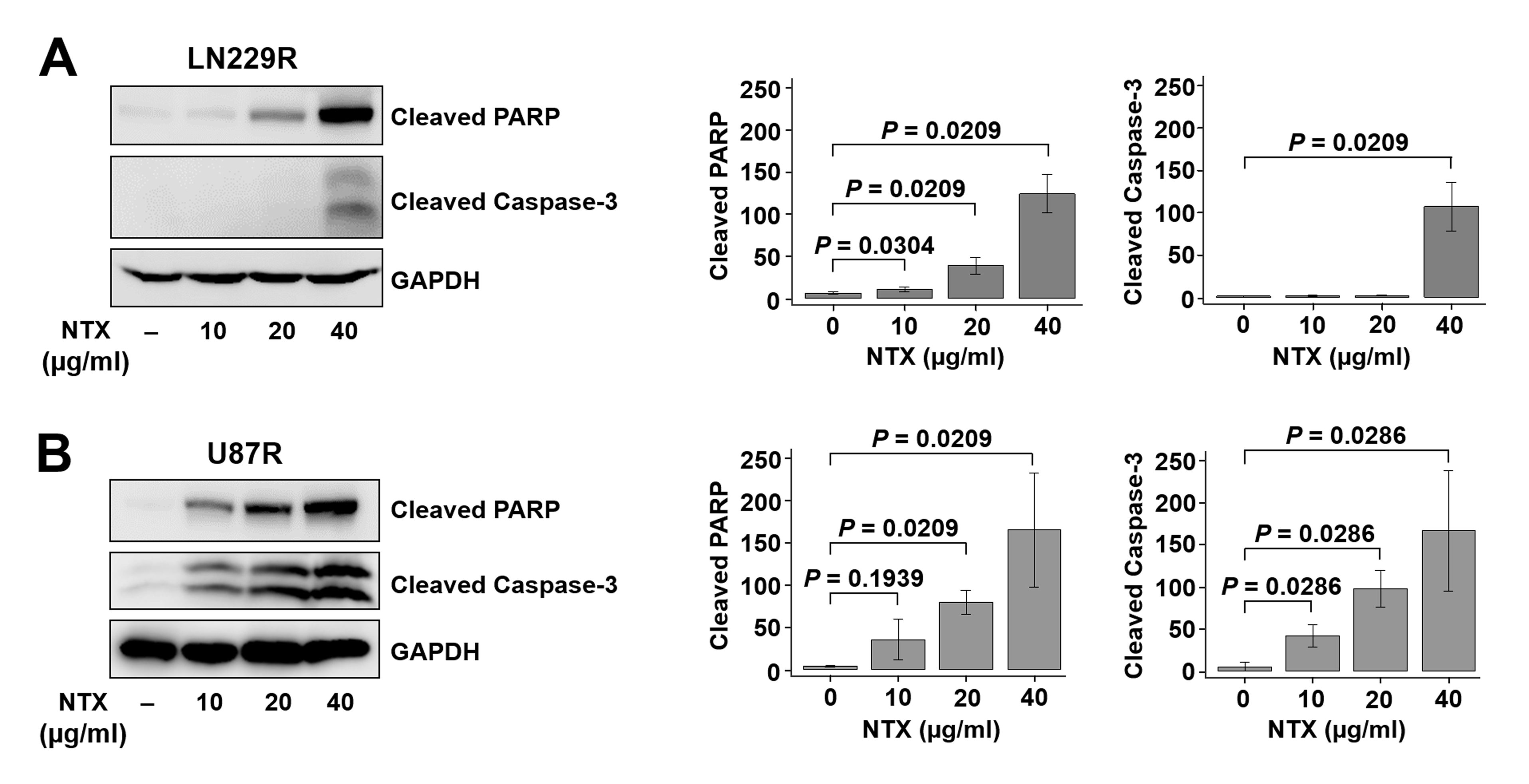


**Figure S2.** Cleaved caspase-3 and PARP expression after NTX treatment in both TMZ-resistant cell lines. **(A)** The increased protein expression level of cleaved PARP with increased concentrations of NTX in LN229R cells confirmed by western blot (full length western blots are provided in Supplementary Fig. S4). However, the expression of cleaved caspase-3 was detected after treatment with 40 µg/ml NTX (*P* = 0.0209) in LN229R cells. **(B)** Increased expression of cleaved caspase-3 and PARP with increased concentrations of NTX in U87R cells confirmed by western blot ((full length western blots are provided in Supplementary Fig. S4). Note: LN229R = TMZ-resistant LN229, and U87R = TMZ-resistant U87.

**
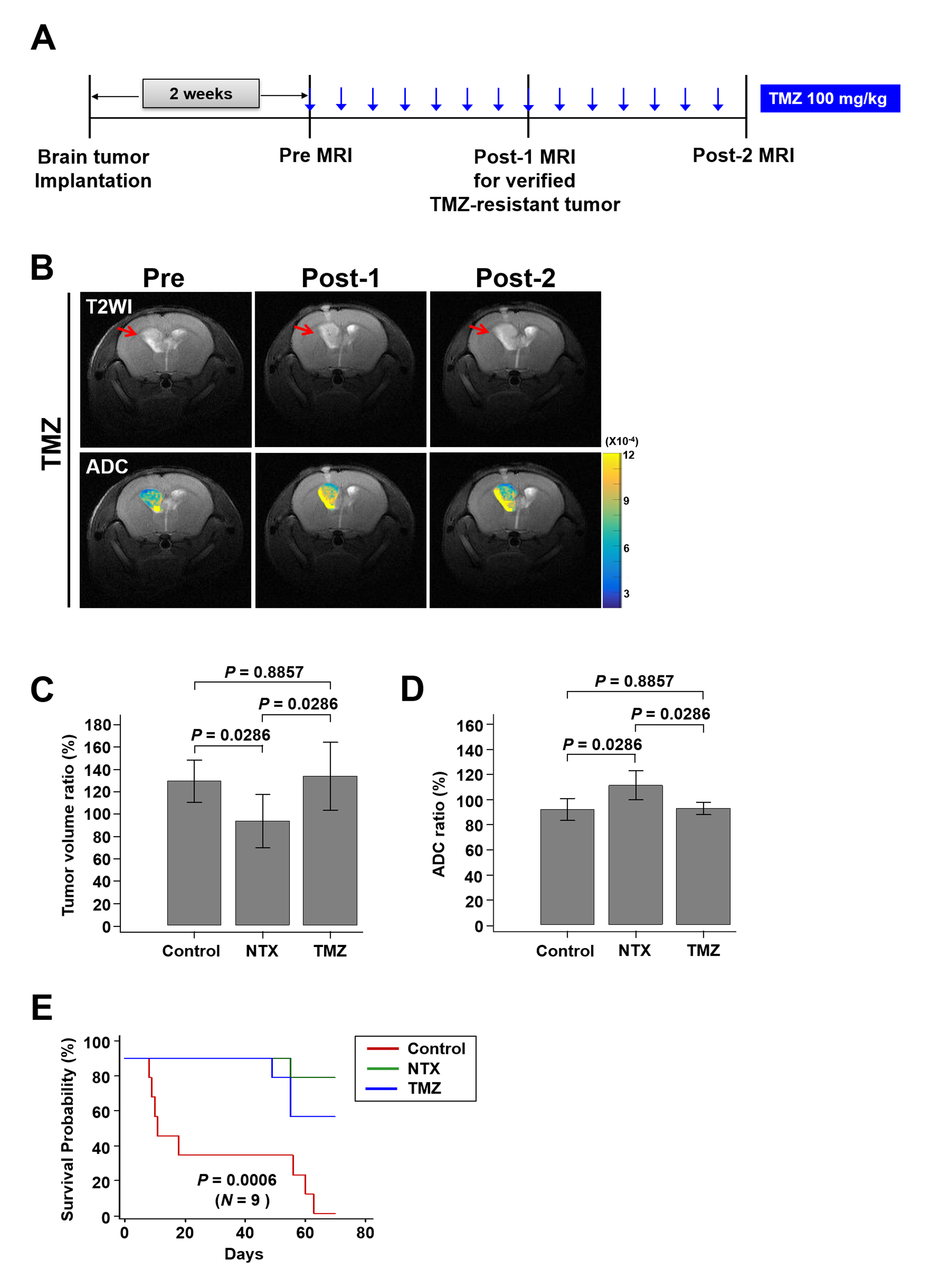
Figure S3.** Short-term *in vivo* MR study and long-term survival analysis in a TMZ-resistant GBM mouse model for the TMZ group.**(A)** The experimental design for the *in vivo* study (n = 4). **(B)** MRI showed anatomical T2WI and ADC maps. **(C)** The tumor volume ratio was significantly increased in the TMZ group compared to the NTX group (*P* = 0.0286). However, there was no significant difference between the tumor volume ratio of the TMZ group and the control group (*P* = 0.8857). **(D)** The ADC ratio was significantly decreased in the TMZ group compared to the NTX group (*P* = 0.0286). However, there was no significant difference between the ADC ratio of the TMZ group and the control group (*P* = 0.8857). **(E)** A significant difference in survival rate was observed among the control, NTX, and TMZ groups [mean, 33.9 (95% CI, 17.1-50.7), 72.8 (95% CI, 68.7-76.9), and 64.3 (95% CI, 59.0-69.7) days; *P* = 0.0006, log-rank test] (*n* = 9 in each group).


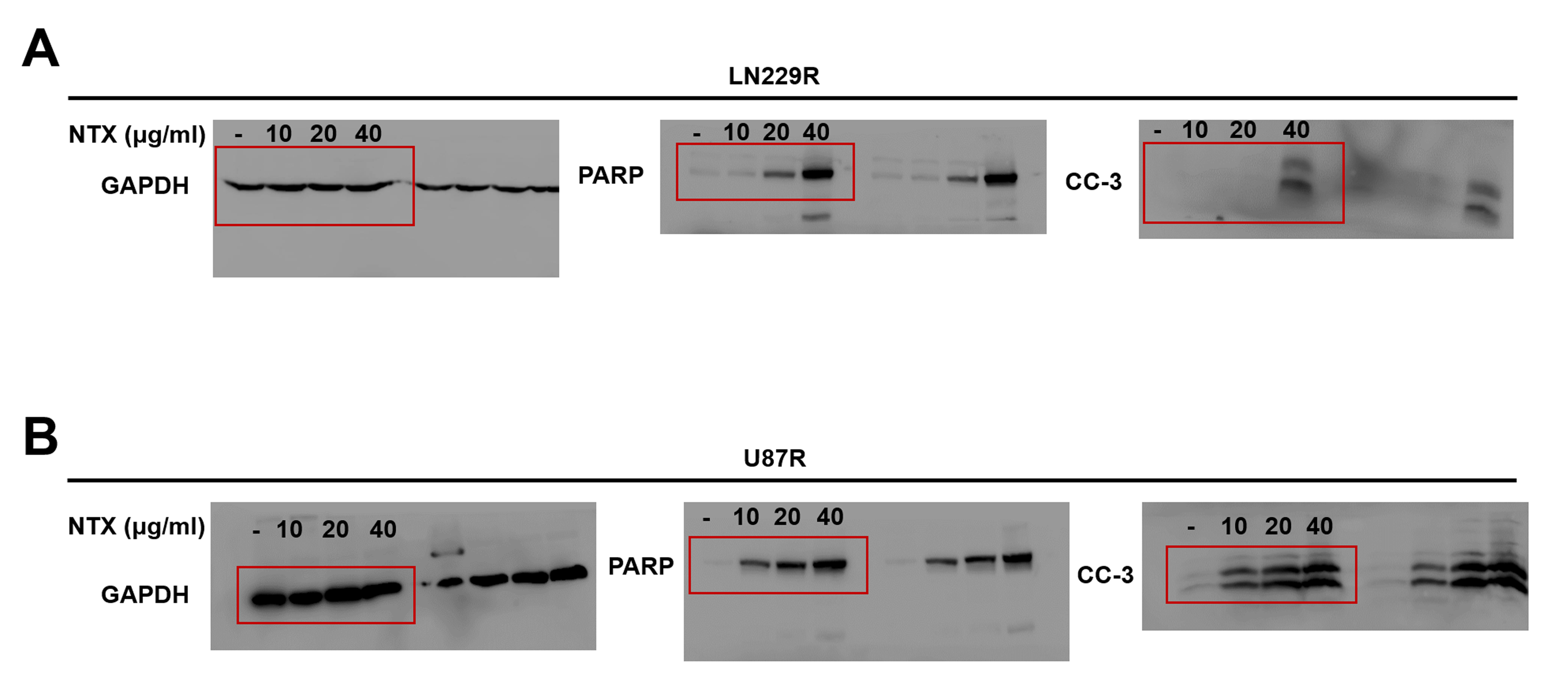


**Figure S4. Full length blots for Figure S2.** (A) LN229R (0, 10 20, 40 μg/ml of NTX) (B) U87R (0, 10 20, 40 μg/ml of NTX). The cropped blots for Figure S2 were marked by red square.

Table

| **Group** | **Control** | **NTX** | **TMZ** |
| --- | --- | --- | --- |
| **Control** | - | 0.06778  (0.01526 - 0.3010) | 0.2262  (0.04947 - 1.0342) |
| **NTX** | 14.7541  (3.3222 - 65.5252) | - | 3.3371  (0.9234 - 12.0609) |
| **TMZ** | 4.4212  (0.9669 - 20.2163) | 0.2997  (0.08291 - 1.0830) | - |

**Supplementary Table S1.** Hazard ratio with 95% confidence interval for the survival of all treatment groups.
